# A very important step: indexation in *Thomson Reuters – Journal Citation Reports*

**DOI:** 10.1590/S1808-86942011000500001

**Published:** 2015-10-22

**Authors:** Fernando Freitas Ganança, Mariana de Carvalho Leal Gouveia, Wilma Terezinha Anselmo-Lima

The *Brazilian Journal of Otorhinolaryngology* (Braz J Otorhinolaryngol), considered the main Brazilian scientific journal in the field of Otorhinolaryngology, has just been indexed in *Thomson Reuters - Journal Citation Reports*, in July of this year. Now, “our” publication is the first Latin-American journal to achieve this much desired goal in the field of Otorhinolaryngology.

This is a reason for much satisfaction and pride, because it is not only an acknowledgement of our journal's scientific quality, but being indexed will make it more visible and accessible to the world community of otorhinolaryngologists, physicians of all other specialties, and other healthcare professionals.

Such achievement has been the result of an effort which started in previous administrations, and the application to the *Thomson Reuters - Journal Citation Reports* was coordinated by João Ferreira de Mello Jr.,MD; Sílvio Caldas Neto, MD; and Regina Martins, MD. The current editorial board continued in this pursue during the first semester of this year, accomplishing this first and important step in the internationalization of the journal of the “Associação Brasileira de Otorrinolaringologia e Cirurgia Cérvico-Facial” (ABORL-CCF, Brazilian Association of Otorhinolaryngology and Neck-Facial Surgery).

The growing number of high-quality papers submitted, the rigorous review and selection of the best papers, the dedication of the editorial board, and the constant support of the ABORL-CCF have made this goal a reality. We would like to thank our Brazilian and international colleagues in the field of Otorhinolaryngology and from other healthcare fields who have contributed with papers and/or with the process of selecting them.

The responsibility of the current and future editorial boards increases with this achievement, because now we will face the challenge of maintaining the high level of the publications and the increased number of citations of the papers published in the *Braz J Otorhinolaryngol*, progressively strengthening its Impact Factor.

We know that, in order to maintain this quality and to attract the submission and publication of good papers, we must continue to improve the efficiency and speed of the editorial process, facilitating the work of authors and revisers.

The editors of the *Braz J Otorhinolaryngol* invite and stimulate authors to submit scientific papers, and we pledge to continue to work so that Brazilian and international specialists dealing with the various topics related to Otorhinolaryngology and Neck-Facial Surgery may continue to receive up-to-date, relevant and reliable information originating from well-managed and ethical studies, which can also contribute to their daily clinical practice.

Sincerely,

